# Genome-wide transcriptional analysis of temperature shift in L. interrogans serovar lai strain 56601

**DOI:** 10.1186/1471-2180-6-51

**Published:** 2006-06-09

**Authors:** Jin-Hong Qin, Yue-Ying Sheng, Zhi-Ming Zhang, Yao-Zhou Shi, Ping He, Bao-Yu Hu, Yang Yang, Shi-Gui Liu, Guo-Ping Zhao, Xiao-Kui Guo

**Affiliations:** 1 School of Life Science/Chemical Engineering, Sichuan University, Chengdu 610041, PR China; 2 Department of Microbiology and Parasitology, Shanghai Jiao Tong University School of Medicine, Shanghai 200025, PR China; 3 National Engineering Center for Biochip at Shanghai, 151 Libing Road, Zhangjiang Hi-Tech Park, Pudong, Shanghai 201203, China

## Abstract

**Background:**

*Leptospira interrogans *is an important mammalian pathogen. Transmission from an environmental source requires adaptation to a range of new environmental conditions in the organs and tissues of the infected host. Several studies have shown that a shift in culture temperature from 28°C to 37°C, similar to that encountered during infection of a host from an environmental source, is associated with differential synthesis of several proteins of the outer membrane, periplasm and cytoplasm. The whole genome of the *Leptospira interrogans serogroup Icterohaemorrhagiae serovar lai type strain #56601 *was sequenced in 2003 and microarrays were constructed to compare differential transcription of the whole genome at 37°C and 28°C.

**Results:**

DNA microarray analyses were used to investigate the influence of temperature on global gene expression in *L. interrogans *grown to mid-exponential phase at 28°C and 37°C. Expression of 106 genes differed significantly at the two temperatures. The differentially expressed genes belonged to nine functional categories: Cell wall/membrane biogenesis genes, hemolysin genes, heat shock proteins genes, intracellular trafficking and secretion genes, two-component system and transcriptional regulator genes, information storage and processing genes, chemotaxis and flagellar genes, metabolism genes and genes with no known homologue. Real-time reverse transcription-PCR assays confirmed the microarray data.

**Conclusion:**

Microarray analyses demonstrated that *L. interrogans *responds globally to temperature alteration. The data delineate the spectrum of temperature-regulated gene expression in an important human pathogen and provide many new insights into its pathogenesis.

## Background

The *leptospires *are motile, helical bacteria constituting a physiologically unique genus of spirochetes that includes the saprophyte *L. biflexa *and the pathogen *L. interrogans*. Leptospirosis is a globally important zoonotic disease caused by pathogenic *Leptospira *species including *L. alexanderi*, *L. borgpetersenii*, *L. interrogans sensu stricto*, it L. kirschneri, *L. noguchii*, *L. santarosai*, *L. weilii*, *L. fainei*, *L. inadai and L. meyeri *[1]. It affects a wide range of mammalian hosts, including humans, horses, dogs, pigs, cattle and wildlife. Because of the large spectrum of animal species that serve as reservoirs, leptospirosis is considered to be the most widespread zoonotic disease [2]. Aside from warm-blooded animals, *leptospires *can also survive in swamps, streams and rivers and alkaline muds and soils [1]. Humans and other animals become infected through contact with urine-contaminated soil and water. When it infects warm-blooded animals, *L. interrogans *must differentially express virulence and other genes at temperatures ranging from roughly 25°C to 37°C. Several in vitro studies have mimicked the temperature shift that *L. interrogans *encounters during infection of a host from an environmental source [3-5]. However, for practical reasons, such studies have been restricted to examination of relatively few proteins.

A more complete analysis of the adaptive responses occurring during the temperature shift will be invaluable for understanding *L. interrogans *transmission, expression of virulence and immune evasion, and for the potential identification of new vaccine candidates. For this purpose, DNA microarrays are being applied to survey globally the adaptive responses to temperature shift in *L. interrogans*. It is now feasible to construct microarrays for analysis of *L. interrogans *because the complete *Leptospira interrogans serogroup Icterohaemorrhagiae serovar lai type strain #56601 *genome is available [6]. In this study, expression of differential genes at 37°C relative to 28°C was studied to elucidate the overall gene expression patterns in *L. interrogans*. The differentially expressed genes found in this study are likely to be expressed differentially during natural mammalian infection and thus provide insights into the infection mechanisms of *L. interrogans*.

## Results and discussion

Entry of *L. interrogans *into a warm-blooded host is usually accompanied by an upshift in temperature. Specific genes are activated or repressed in the bacterial response to temperature elevation [7,8]. To assess gene expression in cultures grown at different temperatures, *L. interrogans *were cultivated to mid-log-phase at 28°C, then passaged into fresh medium incubated either at the original culture temperature or shifted to 37°C (see additional file 4). RNA was isolated from *L. interrogans *that grew well at each temperature. In our present work, two independent cultures were prepared as biological replicates for RNA isolation for each test or reference condition.

### Microarray experiments

Two genome sequences of *L. interrogans serovar Lai *and *L. interrogans serovar Copenhageni *have been released [6,9]. The average nucleotide identity between the two genomes is 95%. The average nucleotide identity between pairs of predicted orthologous protein coding genes is 99%. However, the *serovar Lai *genome has 4727 putative genes annotated while *serovar Copenhageni *has only 3667. It would appear that the *serovar Copenhageni *sequence has fewer putative structural genes than that of *serovar Lai*. However, this difference occurred mainly because the *Copenhageni *sequence contained no predicted coding sequences less than or equal to 150 bp in length that lacked significant homologues. The *serovar Lai *genome has 718 predicted genes of this kind. Genes shorter than 150 bp were indeed expressed according to proteomics analysis (Ren et al., not published). Also, among the genes 180 bp or more in length, 118 are unique to *serovar Lai *and 64 to *serovar Copenhageni *[10]. The 3528 *ORF*s spotted on the microarrays represented most of the genes more than 150 bp in length (excluding the unique genes) in both s*erovars Lai *and *Copenhageni*. Two independent experiments demonstrated that, on average, transcripts of 93% of all genes on the microarrays were detected in the mid-log phase of *L. interrogans*. Data from two independent experiments showed that transcripts of 101 genes were absent under both temperature conditions; these genes seemed not to be expressed at either 28°C or 37°C (see additional file 1).

### Intrachip and interchip reproducibility

To evaluate intrachip and interchip reproducibility, we analyzed the expression values of three copies of all genes at various positions on each of two genechips. After some spots were excluded (SN<2, flag spots), the correlation coefficients R^2 ^of the log_2_(ratio) for three different copies of the gene were 0.824, 0.817, 0.837 for one chip and 0.823, 0.827, 0.878 for another. The correlation coefficient R^2 ^of log_2_(ratio) for the two chips (experiment-to-experiment reproducibility) was 0.665. These results show good chip reproducibility (see also additional file 2).

### Verification of the microarray data

**Figure 1:**
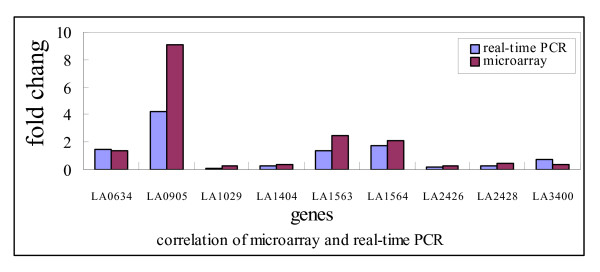
**correlation of microarray and Real-time PCR**. Comparison of transcription measurements by microarray (red bar) and real-time PCR assays (blue bar). The fold change ratios are shown for nine genes at 37°C compared with 28°C.

**Figure 2:**
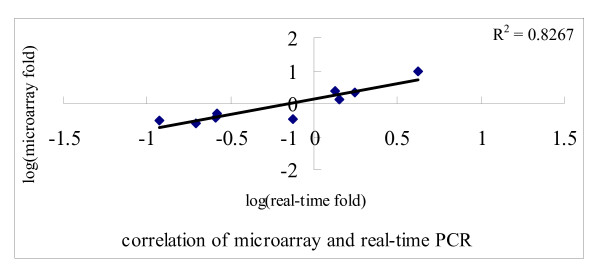
**Comparison of transcription measurements by microarray and real-time PCR assays**. The relative transcriptional levels for the 9 genes listed in Figure 1 were determined by microarray and real-time RT-PCR. The real-time RT-PCR log_2 _values were plotted against the microarray data log_2 _values. The correlation coefficient (R^2^) between the two datasets is 0.8267.

The microarray transcription data were verified by real-time PCR assays of the same sample of 9 genes representing the upregulated, downregulated and unchanged genes (Fig. 1). The log-transformed change in relative quantity of mRNA between each test and reference condition was calculated for each gene. The correlation coefficient R^2 ^between the data obtained by the two techniques was 0.8267 (Fig. 2). Although the fold change in gene expression differed between the microarray and real time PCR results, the general trends were consistent. The real time PCR results therefore corroborated the microarray results but demonstrated the need for confirmation (see also additional file 3).

### Classification of temperature-related genes

**Figure 3:**
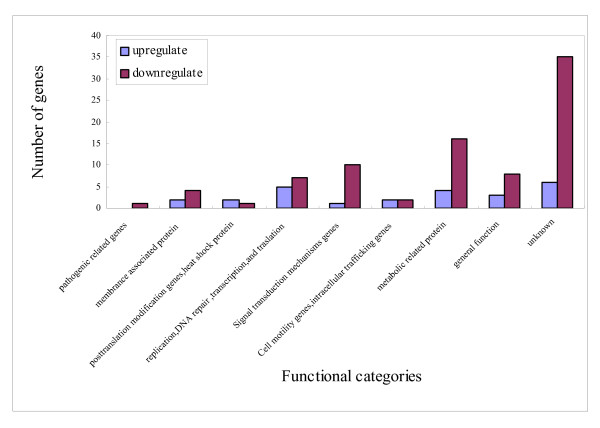
**functional categories**. Genes differentially expressed at 37°C and 28°C, grouped by functional classification according to the NCBI *L. interrogans 56601 *COGs database . Genes were regarded as differentially expressed when the expression levels differed by at least twofold. The number of upregulated (red bars) and downregulated (blue bars) genes in each functional group is shown.

Temperature shift has been reported to alter protein synthesis in *L. interrogans *[3,4,11,12]. In order to characterize these changes at the global genome level, *L. interrogans *was cultured at different temperatures to mid-log-phase and gene expression was quantified. The scanned data generated from Tiffsplit software were imported into GeneSpring 4.0 software for further analysis. Fold change analysis was used to evaluate differential gene expression. The data showed that several genes were induced after temperature shift from 28°C to 37°C; expression of 106 genes was at least twice as high in organisms grown at 37°C as in those grown at 28°C (Fig. 3 and Tables 1, 2). Upregulation was apparent in 24 genes (Table 1) and downregulation in 82 (Table 2). Fewer genes were differentially regulated than in other temperature shift experiments [13,14]. The differentially expressed genes belonged to nine functional categories as shown in Fig 3. Many but not all of these categories contained both upregulated and downregulated genes. Among the upregulated candidates were hsp20 heat shock proteins genes, cell wall/membrane biogenesis genes, intracellular trafficking and secretion genes and information storage and processing genes. Unexpectedly, most of the upregulated genes were of unassigned function. Downregulated genes were more numerous and were represented in more categories than upregulated genes. They include pathogenic genes (including those for chemotaxis and motility), cell wall/membrane biogenesis genes, signal transduction mechanism genes and metabolism genes. The fact that more genes are downregulated at 37°C than at 28°C might partly explain why *L. interrogans *grows more slowly at the higher temperature. Taken together, the data suggest that the composition of the *L. interrogans *proteome is substantially influenced by temperature.

### Heat shock protein genes

In contrast to classical heat shock studies, our observations are based on shifting cultures from 28°C to 37°C and growing them for several days, simulating conditions that would be encountered during the infection of hosts from environmental sources. Typically, two major Hsps, *GroEL *and *DnaK *(members of the Hsp60 and Hsp70 families, respectively), are of considerable importance in the immunology and pathology of various bacterial and parasitic infections. No differences between the expression levels of *DnaK *and *GroEL *in *L. interrogans *maintained at 28°C and those shifted from 28°C to 37°C were detected in our study. The same results in protein expression were also reported by Jarlath E. Nally [4,11]. However, two other small heat shock genes (LA1563 and LA1564) (Table 1) belonging to the Hsp20 family showed increased expression when the organisms were cultured at 37°C for several days. Some studies have shown that low molecular mass heat shock proteins (HSPs) appear to act as molecular chaperones [15,16]. Others have shown that members of the Hsp20 family protect effectively against stress [17]. For example, in *Babesia bovis*, Hsp20 proteins are involved in the cellular response to stress. When the temperature was increased, Hsp20 expression was upregulated [18]. There have been no reports of temperature-related changes in expression of these two proteins in *L. interrogans*. The present result shows that these two Hsp20s play an important role in the response to temperature shift in *L. interrogans*.

### Membrane protein genes

Membrane proteins, especially outer membrane proteins, are critical for understanding the interactions of bacteria with their environments and are the main candidates for protective antigens in extracellular pathogens. Many membrane proteins play an essential role in the development of new immunoprotection and serodiagnosis strategies [19]. Therefore, many studies have been focused on membrane proteins [20-22]. A recent surfaceome study of *Leptospira *showed that the expression of constituents remained unchanged under temperature changes [21]. The microarray data showed the same result. The genes for only six membrane-associated proteins were differentially expressed during culture at 37°C relative to 28°C. Two (LA3927 and LA1203) was upregulated and four (LA2200, LA2248, LA4232 and LA1404) were downregulated at 37°C relative to 28°C. LA3927 is one of two orthologues of the type I secretion TolC protein in *L. interogans*, which is a outer membrane channel protein playing a role in the secretion of extracellular hemolysins and enzymes [10]. Studies on other bacteria have shown that TolC in association with other membrane proteins exports a wide variety of drugs and toxic compounds [23]. In *Vibrio alginolyticu*s, TolC is a stress-responsive protein [12]. It seems that increasing tolC expression in *L. interrogans *may be associated with virulence and with changes in export and other aspects of metabolism in response to the temperature shift. Another three downregulated membrane protein genes with no assigned function should be further studied to establish their physiological role in *L. interrogans*.

### Hemolysins encoded genes

The primary lesion caused by *Leptospira *is damage to the endothelia of small blood vessels, leading to haemorrhage and localized ischaemia in multiple organs. As a consequence, renal tubular necrosis, hepatocellular damage, meningitis and myositis may occur in the infected host [1,24]. Hemolysins may play a fundamental role in this process [24]. Several hemolysin genes have been identified in the *L. interrogans *genome. We therefore determined whether these genes (LA0327, LA0378, LA1027, LA1029, LA1650, LA3050, LA3937 and LA4004) were differentially expressed at 37°C relative to 28°C. Interestingly, one hemolysin gene (LA1029) encoding sphingomyelinase C was downregulated at the higher temperature, but the other hemolysin genes showed unchanged expression. The mechanism of regulation of these genes is not well understood and there have been no previous studies on their response to temperature shifts, so it will be interesting to conduct further studies on their regulation at different temperatures.

### Motility and chemotaxis genes

Motility and chemotaxis are believed to be important in pathogenesis by many bacteria [25]. Motility and chemotaxis responses enable many pathogenic *leptospires *to penetrate host tissue barriers during infection and adapt to a variety of environments and hosts [6,10,26]. Chemotaxis has been extensively studied in the model organism *Escherichia coli*. *E. coli *encodes several chemoreceptors that sense environmental conditions and relay this information to a histidine kinase, CheA, through the coupling protein CheW. CheA phosphorylates the response regulator CheY, which in turn interacts with the flagellar motor in its phosphorylated form, altering both the direction of flagellum rotation and the swimming path of the bacterium [27]. Comparison of the complete sequences suggests that the *L. interrogans *genome contains a relatively large number of motility and chemotaxis genes and has a more complex chemotaxis system than *E. coli *[6,10]. It may be reflects the survival and adaptation of pathogenic Leptospira to a variety of environments and hosts by selected differential expression of different motility and chemotaxis genes. In our study, two chemotaxis genes (LA2426 and LA2427) encoding the chemotaxis protein chew and the methyl-accepting chemotaxis protein were downregulated at 37°C relative to 28°C. Chemotaxis plays multiple roles in the adaptation of a bacterium to its environment, so the changes in these two chemotaxis genes may enable *L. interrogans *to adjust to environmental changes.

### Two-component systems and other regulator genes

The *Leptospira *life cycle requires the ability to respond to a complex array of environmental conditions [1]. One mechanism for adaptation to changing environments is through two-component regulatory systems, a family of proteins that are widely distributed among many bacterial genera [28]. Two-component systems allow specific environmental signals to be detected through a sensor histidine kinase that is usually associated with the cell membrane. In many cases, signaling through a single two-component system results in a coordinated change in expression of multiple genes, the products of which play a role in adaptation to a particular environment [29]. This is the most common type of signal transduction system in bacteria and controls such diverse processes as gene expression, sporulation and chemotaxis [30]. In pathogenic bacteria, two-component regulatory systems can also control the up- and down-regulation of different virulence determinants [29]. In our study, LA2549, a two-component sensor, was upregulated and three two-components (LA2423, LA3357 and LA4104) were downregulated at 37°C relative to 28°C. It is unknown whether these two-components regulate virulence production or only adaptation to the temperature shift, which is disadvantageous for organism growth. These changes in gene regulation might enable the organism to adapt to the hostile environment of the host. In addition to the two-component systems, one anti-sigma factor antagonist gene, two cyclic nucleotide genes containing the GGDEF motif and a GAF domain regulator gene were also down-regulated at 37°C relative to 28°C. Cyclic nucleotides appear to have a major regulatory role in *Leptospira *species [6,9,10]. GGDEF-domain proteins are more commonly found in non-obligate parasitic bacteria than in obligate parasites, indicating their importance in responding to environmental signals [9]. GAF is a cGMP binding domain. *L. interrogans *may respond to the temperature shift through these regulators, thus controlling other physiological changes that are important in adapting to the environment.

### Energy and metabolism genes

Bacteria respond quickly to environmental stimuli, so energy production and metabolism adjust rapidly to new growth conditions. Indeed, many genes related to these processes were upregulated and downregulated by the temperature shift from 28°C to 37°C, as shown in Table 1 and Table 2.

## Conclusion

In this study, cDNA microarrays covering 3528 genes were used to investigate temperature shift adaptation by means of whole genomic transcription analysis. This is the first study of whole genomic transcription using the *L. interrogans *cDNA-genechip based on the complete sequence.

Several global transcription analyses of bacterial responses to growth temperature variation have been published; e.g. *E. coli *[31], g*roup A Streptococcus *[14], *B. subtilis *[32], *Campylobacter jejuni *[33], *Borrelia burgdorfer *[34] and *Mycoplasma pneumoniae *[35]. Although temperature-regulated bacterial gene expression has been well described in *L. interrogans *[3,4,11], our study delineates global gene expression changes in this organism in response to temperature changes.

Bacteria use multiple molecular strategies to alter gene expression in response to temperature change. Our microarray analyses demonstrated that *L. interrogans *responds globally to temperature alteration. Temperature-induced genes include heat shock proteins genes, Cell wall/membrane biogenesis genes, virulence genes, regulatory genes and unidentified proteins. Our data demonstrate that *L. interrogans *has the ability to alter gene transcription extensively in response to temperature during infection. Importantly, many of the genes that are differentially regulated in response to growth temperature encode proteins of unknown function, and thereby provide additional avenues for pathogenesis research.

## Methods

### Bacterial strain, medium, and growth

Isolates of *Leptospira interrogans *(*serogroup Icterohaemorrhagiae, serovar lai, type strain 56601*) were obtained from the Institute for Infectious Disease Control and Prevention (IIDC), Beijing, China. *L. interrogans *was grown in liquid Ellinghausen-McCullough-Johnson-Harris (EMJH) medium at 28°C under aerobic conditions to mid-log-phase and then shifted to fresh EMJH medium incubated at 28°C or 37°C under aerobic conditions. Only mid-log-phase cultures at a mean density of 10^6^/ml in 100 ml were used in gene expression analysis experiments. The cells were harvested by centrifugation at 10,000 g for 10 min at 4°C.

### RNA isolation

For each condition (37°C and 28°C), total RNA was extracted from two independent replicates. Cells were harvested and the complete RNAs were extracted using Trizol reagent (Invitrogen) according to the manufacturer's protocol. Contaminating DNA was digested with RQ1 RNase-free DNase (Promega Corp.). The treated RNAs were purified with a QIAGEN RNeasy Kit (QIAGEN). RNA quality was monitored by agarose gel electrophoresis, and the quantity was determined spectrophotometrically (Ependorf).

### Microarray hybridization

Arrays of whole L. interrogans genome PCR products were based on the sequenced genomes of *Leptospira interrogans serogroup Icterohaemorrhagiae serovar lai type strain #56601*. Of 4,727 total predicted genes, 3700 were incorporated (excluding the 1027 ORFs that are unique or are 150 bp or less in length). Of these 3700 genes, PCRs for 172 consistently failed to yield satisfactory results (no product, product of the wrong size, multiple bands or faint bands), even after redesigned primers were used in the amplification reaction. Thus, 3528 ORFs were correctly amplified. PCR products were spotted on to poly-lysine-coated glass microarray slides with Genemachine. Probes were printed in triplicate on the slides as described in the manual. Each test RNA (10 μg, cultured at 37°C) and reference RNA (10 μg, cultured at 28°C) was labeled with Cy3 or Cy5, respectively, by reverse transcription using Superscript (Invitrogen). The unincorporated dye was removed using a QIAquick Nucleotide Removal Kit (QIAGEN) as specified by the manufacturer's protocol. Samples were hybridized competitively under coverslips to the microarray slides at 42°C for 16 h, and then washed as described in the manual. Hybridization experiments were performed in duplicate using cDNA derived from four different cultures of *L. interrogans *(two grown at 37°C, two at 28°C).

### Data analysis

The hybridization slides were scanned and analyzed by Tiffsplit (Agilent) to calculate the signal intensities and to determine the presence or absence of each open reading frame. The microarrays were then normalized, and their backgrounds were defined using GeneSpring 4.0 (Silicon Genetics). The GeneSpring software was used to analyze the transcription patterns further. To identify genes with significantly altered expression levels for further analysis, cutoff values for expression level ratios 2.0 and 0.5 were used to filter genes with changes (n-fold) greater than ± 2.0 in two independent biological samples, even though a 1.5-fold cutoff has recently been reported as biologically significant [14,33,36]. Student's t test/analysis of variance was used to compare the mean expression levels of the test and reference samples. Genes with significant differential expression levels (P < 0.05) were selected.

For intrachip and interchip reproducibility analysis, flagged spots or SN<2 spots were excluded. The coefficients of three spots in same chip for each gene were calculated to estimate intrachip reproducibility using Microsoft Excel. The coefficient of an average of two independent biological samples was calculated to estimate interchip reproducibility.

For functional gene categories, most ORFs were taken from Genbank accession numbers NC004342 and NC004343[37]. For annotation in GenBank as hypothetical and conserved proteins, Cluster of Orthologous Genes (COG) descriptions in NCBI were used [37]. Blast [38] was also used to identify whether homologues were present in other bacteria. Differentially transcribed genes were classified into functional groups by COG classification when available. Genes without COG classification were categorized by their GenBank annotations.

### Real-time quantitative PCR (qPCR)

The RNA samples subjected to microarray analysis were also used to produce cDNA by reverse transcription using Superscript α (Invitrogen) to confirm changes in the expression of selected genes. qPCR was performed using the cDNAs as templates with a Roche real-time PCR machine (Roche) as described [39], using the SYBR green dye and Invitrogen kit (Invitrogen) according to the manufacture's protocol. For each amplification run, the calculated threshold cycle (Ct) for each gene amplicon was normalized to the C_t _of the 16S rRNA gene amplified from the corresponding sample before the gene fold and relative changes were calculated as described [39,40].

## Competing interests

The author(s) declare that they have no competing interests.

## Authors' contributions

JHQ and XKG designed the research project. JHQ, ZMZ and YYS constructed the microarray. JHQ, ZMZ and PH performed the microarray study and analyzed the data. JHQ, YY and BYH coordinated the Leptospira culture. JHQ and XKG drafted the manuscript. GPZ and SGL participated in the design of the study and helped to draft the manuscript. All authors contributed in the writing and preparation of the manuscript. All authors read and approved manuscript.

## Supplementary Material

Additional File 1 **Microsoft Excel document, absent genes in two microarrays**. This file and dataset contains 101 genes that that were not expressed whatever conditions (28°C and 37°C) in our experiment. Click here for file

Additional File 2 **Microsoft Excel document, all genes contained in the two microarrays**. This file provides a list of raw data of all the genes contained in the microarrays. Click here for file

Additional File 3 Microsoft Excel document, nine genes contained in the microarrays and Real-time PCR. Click here for file

Additional File 4 Microsoft Excel document, raw data and growth curve of *L. interrogans *cultured at 37°C and 28°. Click here for file

## Figures and Tables

**Table 1:** Genes upregulated at least twofold at 37°C relative to 28°C

Gene ID	Gene function and name	Fold change in	
		Expt1	Expt2
Cell wall/membrane biogenesis genes			
LA1203	alginate o-acetyltransferase	2.393	2.084
LA3927	Outer membrane protein tolC precursor	3.337	3.817
Posttranslational modification, heat shock proteins genes			
LA1563	class II heat shock protein (HSP20)	2.439	4.233
LA1564	class II heat shock protein (HSP20)	2.142	2.859
replication, DNA repair, transcription and translation genes			
LA0877	Probable RNA polymerase ECF-type sigma factor	4.704	4.752
LA0878	DshA protein	3.305	3.076
LA2204	hypothetical protein	2.027	3.693
LA3749	hypothetical protein	2.200	4.771
LA0065	DNA-damage-inducible protein F	2.356	2.028
Signal transduction mechanism genes			
LA2549	Sensory transduction histidine kinase	3.174	2.309
intracellular trafficking and secretion genes			
LA0905	hypothetical protein	9.108	7.718
LA3927	Outer membrane protein tolC precursor	3.337	3.817
metabolism genes			
LA0633	Probable peptide transporter permease	2.441	2.025
LA3498	Phosphate transport system protein phoU	2.934	4.779
LA0430	hypothetical protein	7.594	4.995
LA1889	Putative 1-aminocyclopropane-1-carboxylate deaminase	2.692	2.519
general function			
LA0297	CBS domain protein	2.464	2.200
LA0450	conserved hypothetical protein	2.631	2.244
LA0700	hypothetical protein	3.174	2.309
unknown			
LA2440	hypothetical protein	2.309	2.956
LA2727	unknown protein	2.212	2.030
LA0430	hypothetical protein	7.594	4.995
LA1809	conserved hypothetical protein	2.155	2.882
LA2465	unknown protein	2.284	3.392
LA2764	hypothetical protein	6.620	7.058
LA4191	hypothetical protein	2.063	2.936

**Table 2:** Genes downregulated at least twofold at 37°C relative to 28°

Gene ID	Gene function and name	Fold change in	
		Expt1	Expt2
pathogenic genes			
LA1029	Sphingomyelinase C precursor	0.318	0.181
Cell wall/membrane biogenesis genes			
LA2248	putative outer membrane protein	0.473	0.405
LA2200	putative N-acetylmuramoyl-L-alanine amidase	0.20	0.278
LA4232	conserved hypothetical protein	0.413	0.383
LA1404	putative outer membrane protein	0.394	0.350
Posttranslational modification, heat shock proteins genes			
LA3562	conserved hypothetical protein	0.457	0.450
replication, DNA repair, transcription and translation genes			
LB112	putative regulatory protein contains GAF domain	0.491	0.395
LB367	hypothetical protein	0.414	0.285
LA2774	conserved hypothetical protein	0.380	0.423
LA4236	MutS-like mismatch repair protein, ATPases	0.297	0.265
LA3011	Cell division protein ftsK homologue	0.397	0.469
LA0937	hypothetical protein	0.447	0.429
LA3419	DNA-directed RNA polymerase, beta subunit	0.327	0.37
Signal transduction mechanism genes			
LA1483	GGDEF domain protein	0.481	0.456
LA2423	two-component response regulator	0.479	0.471
LA2426	Methyl-accepting chemotaxis protein	0.260	0.197
LA2427	Chemotaxis protein chew	0.450	0.335
LA2434	probable anti-sigma factor antagonist	0.483	0.378
LA3107	unknown protein	0.185	0.153
LB112	putative regulatory protein contains GAF domain	0.491	0.395
LA3950	hypothetical protein	0.462	0.465
LA3357	Sensory transduction histidine kinase	0.465	0.474
LA4104	two-component hybrid sensor and regulator	0.455	0.450
Chemotaxis and flagellar genes			
LA2426	Methyl-accepting chemotaxis protein	0.260	0.197
LA2427	Chemotaxis protein chew	0.450	0.335
metabolism genes			
LA0106	long-chain-fatty-acid CoA ligase	0.463	0.442
LA0828	Acetyl-CoA acetyl transferases	0.469	0.332
LA1430	3-oxoacyl-[acyl-carrier-protein] synthase	0.493	0.356
LA2008	hydrolipoamide acetyltransferase component of pyruvate dehydrogenase complex	0.469	0.332
LA2009	pyruvate dehydrogenase E1 component, beta subunit	0.451	0.371
LA2010	pyruvate dehydrogenase E1 component, alpha subunit	0.486	0.435
LA1485	mRNA-binding proteins	0.278	0.436
LA2724	hypothetical protein	0.116	0.320
LA3168	hypothetical protein	0.280	0.230
LA4176	conserved hypothetical protein	0.480	0.448
LA4233	hypothetical protein	0.294	0.190
LA3312	glyoxalase	0.497	0.436
LA3628	anthranilate synthase component I	0.364	0.403
LA3998	cholesterol oxidase	0.381	0.358
LA4349	putative peptidase	0.437	0.349
LB093	probable long-chain-fatty-acid – CoA ligase	0.338	0.296
general function			
LA0827	hypothetical protein	0.332	0.410
LA1920	RNA-binding protein	0.301	0.0823
LA2724	hypothetical protein	0.116	0.320
LA3168	hypothetical protein	0.280	0.230
LA3400	processing proteinase	0.350	0.491
LA3998	cholesterol oxidase	0.381	0.358
LB265	conserved hypothetical protein	0.369	0.376
LA3584	TPR-repeat-containing protein	0.0539	0.159
unknown			
LA0091	conserved hypothetical protein	0.444	0.441
LA2259	conserved hypothetical protein	0.362	0.358
LA3196	conserved hypothetical protein	0.239	0.200
LA1031	hypothetical protein	0.223	0.476
LA0278	hypothetical protein	0.0698	0.376
LA1403	hypothetical protein	0.252	0.233
LA1468	conserved hypothetical protein	0.286	0.316
LA1572	conserved hypothetical protein	0.391	0.359
LA1910	hypothetical protein	0.452	0.495
LA1973	hypothetical protein	0.399	0.398
LA2020	hypothetical protein	0.260	0.177
LA2624	hypothetical protein	0.462	0.472
LA2859	unknown protein	0.401	0.358
LA2946	hypothetical protein	0.370	0.103
LA3036	hypothetical protein	0.363	0.480
LA3083	hypothetical protein	0.412	0.272
LA3344	hypothetical protein	0.123	0.184
LA3452	hypothetical protein	0.326	0.378
LA4030	hypothetical protein	0.469	0.350
LA4046	hypothetical protein	0.490	0.450
LA4282	hypothetical protein	0.454	0.420
LB032	hypothetical protein	0.422	0.485
LB217	hypothetical protein	0.442	0.324
LB217	hypothetical protein	0.324	0.442
LB243	hypothetical protein	0.375	0.448
LB316	unknown protein	0.345	0.290
LA1178	hypothetical protein	0.188	0.432
LA1306	hypothetical protein	0.395	0.388
LA1952	hypothetical protein	0.440	0.419
LA2013	hypothetical protein	0.262	0.243
LA2624	hypothetical protein	0.111	0.158
LA2720	hypothetical protein	0.190	0.458
LA2839	hypothetical protein	0.233	0.346
LA4095	hypothetical protein	0.377	0.341
LA1800	Hypothetical protein	0.485	0.242

## References

[B1] Faine S, Adler B, Bolin C, Perolat P (1999). Leptospira and leptospirosis.

[B2] Levett PN (2001). Leptospirosis. Clin Microbiol Rev.

[B3] Jarlath E, Nally SA, Timoney JohnF (2001). Molecular Characterization of Thermoinduced Immunogenic Proteins Q1p42 and Hsp15 of Leptospira interrogans. Infection and Immunity.

[B4] Nally JE, Timoney JF, Stevenson B (2001). Temperature-regulated protein synthesis by Leptospira interrogans. Infect Immun.

[B5] Cullen PA, Cordwell SJ, Bulach DM, Haake DA, Adler B (2002). Global analysis of outer membrane proteins from Leptospira interrogans serovar Lai. Infect Immun.

[B6] Ren SX, Fu G, Jiang XG, Zeng R, Miao YG, Xu H, Zhang YX, Xiong H, Lu G, Lu LF, Jiang HQ, Jia J, Tu YF, Jiang JX, Gu WY, Zhang YQ, Cai Z, Sheng HH, Yin HF, Zhang Y, Zhu GF, Wan M, Huang HL, Qian Z, Wang SY, Ma W, Yao ZJ, Shen Y, Qiang BQ, Xia QC, Guo XK, Danchin A, Saint GI, Somerville RL, Wen YM, Shi MH, Chen Z, Xu JG, Zhao GP (2003). Unique physiological and pathogenic features of Leptospira interrogans revealed by whole-genome sequencing. Nature.

[B7] Yura TKN (1999). Regulation of the heat-shock response. Curr Opin Microbiol.

[B8] Gophna UEZR (2003). Virulence and the heat shock response. Int J Med Microbiol 2.

[B9] Nascimento AL, Verjovski-Almeida S, Van Sluys MA, Monteiro-Vitorello CB, Camargo LE, Digiampietri LA, Harstkeerl RA, Ho PL, Marques MV, Oliveira MC, Setubal JC, Haake DA, Martins EA (2004). Genome features of Leptospira interrogans serovar Copenhageni. Braz J Med Biol Res.

[B10] Nascimento AL, Ko AI, Martins EA, Monteiro-Vitorello CB, Ho PL, Haake DA, Verjovski-Almeida S, Hartskeerl RA, Marques MV, Oliveira MC, Menck CF, Leite LC, Carrer H, Coutinho LL, Degrave WM, Dellagostin OA, El-Dorry H, Ferro ES, Ferro MI, Furlan LR, Gamberini M, Giglioti EA, Goes-Neto A, Goldman GH, Goldman MH, Harakava R, Jeronimo SM, Junqueira-de-Azevedo IL, Kimura ET, Kuramae EE, Lemos EG, Lemos MV, Marino CL, Nunes LR, de-Oliveira RC, Pereira GG, Reis MS, Schriefer A, Siqueira WJ, Sommer P, Tsai SM, Simpson AJ, Ferro JA, Camargo LE, Kitajima JP, Setubal JC, Van Sluys MA (2004). Comparative genomics of two Leptospira interrogans serovars reveals novel insights into physiology and pathogenesis. J Bacteriol.

[B11] Stamm LV, Gherardini FC, Parrish EA, Moomaw CR (1991). Heat shock response of spirochetes. Infect Immun.

[B12] Xu CWS, Ren H, Lin X, Wu L, Peng X (2005). Proteomic analysis on the expression of outer membrane proteins of Vibrio alginolyticus at different sodium concentrations. Proteomics.

[B13] Han Y, Zhou D, Pang X, Song Y, Zhang L, Bao J, Tong Z, Wang J, Guo Z, Zhai J, Du Z, Wang X, Zhang X, Wang J, Huang P, Yang R (2004). Microarray analysis of temperature-induced transcriptome of Yersinia pestis. Microbiol Immunol.

[B14] Smoot LM, Smoot JC, Graham MR, Somerville GA, Sturdevant DE, Migliaccio CA, Sylva GL, Musser JM (2001). Global differential gene expression in response to growth temperature alteration in group A Streptococcus. Proc Natl Acad Sci USA.

[B15] Kanno Y, Matsuno H (2006). The Possibility of Novel Antiplatelet Peptides: The Physiological Effects of Low Molecular Weight HSPs on Platelets. Curr Pharm Des.

[B16] Horwitz J (1992). Alpha-crystallin can function as a molecular chaperone. Proc Natl Acad Sci USA.

[B17] Yan-hui ZHU T-mM, Xian WANG (2005). Gene transfer of heat-shock protein 20 protects against ischemia/reperfusion injury in rat hearts. Acta Pharmacologica Sinica.

[B18] Lee S, Carson K, Rice-Ficht A, Good T (2005). Hsp20, a novel alpha-crystallin, prevents Abeta fibril formation and toxicity. Protein Sci.

[B19] Zhang XY, Yu Y, He P, Zhang YX, Hu BY, Yang Y, Nie YX, Jiang XG, Zhao GP, Guo XK (2005). Expression and comparative analysis of genes encoding outer membrane proteins LipL21, LipL32 and OmpL1 in epidemic leptospires. Acta Biochim Biophys Sin (Shanghai).

[B20] Nally JE, Whitelegge JP, Aguilera R, Pereira MM, Blanco DR, Lovett MA (2005). Purification and proteomic analysis of outer membrane vesicles from a clinical isolate of Leptospira interrogans serovar Copenhageni. Proteomics.

[B21] Cullen PA, Xu X, Matsunaga J, Sanchez Y, Ko AI, Haake DA, Adler B (2005). Surfaceome of Leptospira spp. Infect Immun.

[B22] Haake DA, Matsunaga J (2005). Leptospiral membrane proteins – variations on a theme?. Indian J Med Res.

[B23] Baucheron SMC, Praud K, Chaslus-Dancla E, Cloeckaert A (2005). TolC but not AcrB is essential for multidrug-resistant Salmonella enterica serotype Typhimurium colonization of chicks. J Antimicrob Chemother.

[B24] Yi-xuan ZHANG YG, Bo BI, Jian-yong HE, Chun-fu WU, Xiao-kui GUO, Guo-ping ZHAO (2005). Identification and classification of all potential hemolysin encoding genes and their products from Leptospira interrogans serogroup Icterohaemorrhagiae serovar Lai. Acta Pharmacologica Sinica.

[B25] Motaleb MAMM, Li C, Bakker RG, Goldstein SF, Silversmith RE, Bourret RB, Charon NW (2005). CheX Is a Phosphorylated CheY Phosphatase Essential for Borrelia burgdorferi Chemotaxis. J Bacteriol.

[B26] Charon NW, Goldstein SF (2002). Genetics of motility and chemotaxis of a fascinating group of bacteria: the spirochetes. Annu Rev Genet.

[B27] Armitage JP (1999). Bacterial tactic responses. Adv Microb Physiol.

[B28] Parkinson JS (1993). Signal transduction schemes of bacteria. Cell.

[B29] Jiang SM, Cieslewicz MJ, Kasper DL, Wessels MR (2005). Regulation of virulence by a two-component system in group B streptococcus. J Bacteriol.

[B30] Novick RP (2003). Autoinduction and signal transduction in the regulation of staphylococcal virulence. Mol Microbiol.

[B31] Richmond CS, Glasner JD, Mau R, Jin H, Blattner FR (1999). Genome-wide expression profiling in Escherichia coli K-12. Nucleic Acids Res.

[B32] Helmann JD, Wu MF, Kobel PA, Gamo FJ, Wilson M, Morshedi MM, Navre M, Paddon C (2001). Global transcriptional response of Bacillus subtilis to heat shock. J Bacteriol.

[B33] Stintzi A (2003). Gene expression profile of Campylobacter jejuni in response to growth temperature variation. J Bacteriol.

[B34] Ojaimi C, Brooks C, Casjens S, Rosa P, Elias A, Barbour A, Jasinskas A, Benach J, Katona L, Radolf J, Caimano M, Skare J, Swingle K, Akins D, Schwartz I (2003). Profiling of temperature-induced changes in Borrelia burgdorferi gene expression by using whole genome arrays. Infect Immun.

[B35] Weiner J, Zimmerman CU, Gohlmann HW, Herrmann R (2003). Transcription profiles of the bacterium Mycoplasma pneumoniae grown at different temperatures. Nucleic Acids Res.

[B36] Hughes TR, Marton MJ, Jones AR, Roberts CJ, Stoughton R, Armour CD, Bennett HA, Coffey E, Dai H, He YD, Kidd MJ, King AM, Meyer MR, Slade D, Lum PY, Stepaniants SB, Shoemaker DD, Gachotte D, Chakraburtty K, Simon J, Bard M, Friend SH (2000). Functional discovery via a compendium of expression profiles. Cell.

[B37] http://www.ncbi.nlm.nih.gov/entrez/query.fcgi?db=genome&cmd=Retrieve&dopt=Overview&list_uids=258.

[B38] http://www.ncbi.nlm.nih.gov/BLAST/.

[B39] Schmittgen TD, Zakrajsek BA, Mills AG, Gorn V, Singer MJ, Reed MW (2000). Quantitative reverse transcription-polymerase chain reaction to study mRNA decay: comparison of endpoint and real-time methods. Anal Biochem.

[B40] Livak KJ, Schmittgen TD (2001). Analysis of relative gene expression data using real-time quantitative PCR and the 2(-Delta Delta C(T)) Method. Methods.

